# Risk Perception of Military Firefighters and Brigades in Relation to Exposure to Smoke from Forest Fires in Brazil

**DOI:** 10.3390/toxics14050431

**Published:** 2026-05-13

**Authors:** Fausto Jaime Miranda de Araujo, Eloisa Dutra Caldas

**Affiliations:** Laboratory of Toxicology, Faculty of Health Sciences, University of Brasília, Brasilia 70910-900, DF, Brazil; faustosst@hotmail.com

**Keywords:** risk perception, wildfires, military firefighters, forest brigades, respiratory protection equipment

## Abstract

Firefighters and forest brigades engaged in wildfire suppression are routinely exposed to smoke containing toxic compounds that pose acute and chronic health risks, and it is important to understand how they perceive these risks during their work. This study aimed to evaluate health risk perception among military firefighters and contracted forest brigades in the Federal District, Brazil, the use of respiratory protection equipment (RPE), and institutional support. A questionnaire was administered to 150 firefighters and 22 brigades in 2023 and 2024. Most respondents were between 30 and 40 years old, with firefighters having a significantly higher education level than brigades (*p* < 0.0001). Most were concerned about smoke exposure and recognized its high health risk, including respiratory diseases and cancer, with brigades showing a higher risk perception than firefighters (*p* < 0.0001). Despite this high perceived risk, about 80% of firefighters and 86% of brigades reported not using RPE, mainly because it was not provided by their institutions (according to 53.8% of firefighters and 73.7% of brigades). The level of concern about wildfire smoke among participants correlated positively with age, years of experience, perceived necessity of RPE, and willingness to use it if provided. Firefighters rated their institution’s performance on occupational health and safety significantly less positively than brigades (*p* < 0.0001). The results of this study demonstrated that the lack of preventive and protective practices is not due to low risk perception, but rather to institutional failures in guidance, support, and provision of RPE.

## 1. Introduction

Firefighters face daily occupational hazards, with smoke exposure among the most significant [1,2]. Wildfire smoke is composed mainly of CO_2_, water vapor, particulate matter, and several toxic compounds, including polycyclic aromatic hydrocarbons (PAHs), benzene, toluene, ethylbenzene, xylenes, formaldehyde, and acrolein [2,3,4,5]. The levels of benzene in five prescribed in France ranged from 12 to 54 mg/m^3^, which could already result in headaches, lassitude and weakness [6]. These pollutants are associated with respiratory and cardiovascular diseases and cancer following prolonged exposure, which is common during wildfire suppression [7,8,9].

Cherry et al. [7] reported the results of a cohort study to investigate the health effects of 12,731 wildland firefighters who had worked for the Alberta Fire Department (Canada) for at least 1 year. The risk of chronic obstructive pulmonary disease, pneumonia, and asthma increased with cumulative firefighting hours across multiple fire seasons. Also in Canada, Sritharan et al. [8] found that firefighters (N = 13,642) had increased risk of prostate cancer (HR = 1.43, 95% CI: 1.31–1.57), colon cancer (HR = 1.39, 95% CI: 1.19–1.63), and skin melanoma (HR = 2.38, 95% CI: 1.99–2.84). Esteves et al. [10] showed that firefighting activities led to a significant increase in both primary and oxidative DNA damage by 22–23% among Portuguese firefighters. Furthermore, firefighting activities, including exposure to smoke components, were reclassified by the International Agency for Research on Cancer (IARC) in 2022 from Group 2B (possibly carcinogenic) to Group 1 (carcinogenic to humans), based on sufficient evidence for mesothelioma and bladder cancer [11]. This effect is mainly due to benzene and benzo[*a*]pyrene (one of the PAH), which are also classified as carcinogenic to humans [11].

Risk is omnipresent in human life, and its perception cannot be separated from the observer, as risk does not exist in itself but is mentally constructed [12]. Individuals perceive situations as safe or risky depending on the context, and cultural and organizational factors [13]. Understanding this perception is essential for developing interventions that promote changes in occupational routines and health protection [14].

Studies investigating firefighters’ risk perception have been conducted worldwide, including in Latin America. Louzado-Feliciano et al. [15] characterized Dominican firefighters’ perceptions and attitudes regarding occupational cancer risk and prevention practices, and in Ecuador, more than 60% of firefighters consider their occupation to pose a high risk [16]. Fialho et al. [17] concluded that Portuguese firefighters with fewer years of service have lower risk perception, and those with higher perception tend to adopt safer behaviors. The main cognitive risk perceptions related to ergonomic factors, night or prolonged shifts, inhalation of chemical agents, and thermal stress, while emotional risk perceptions were mainly associated with chemical agents, stress, and ergonomic risks [17]. In the only published study involving Brazilian military firefighters, 92.4% of participants reported at least one symptom after exposure, primarily headache, eye irritation, and cough [18].

Studies assessing Brazilian firefighters’ risk perception are limited, and those with the brigades are nonexistent. The main objectives of this study are to understand the risk perception of military firefighters and contracted forest brigades in the Federal District, Brazil, regarding smoke exposure, its health implications, and the use of RPE. To the best of our knowledge, this is the first study to assess the risk perception of contracted forest brigades and compare it to that of firefighters.

## 2. Methods

### 2.1. Study Design and Setting

The study was conducted through a 20-question questionnaire administered to firefighters from the Military Fire Department of the Federal District (CBMDF) and contracted forest brigades from the Brasília Environmental Institute (IBRAM). Convenience sampling was used, i.e., professionals involved in wildfire prevention and suppression activities at both institutions were approached at random and asked to participate. The questionnaire was applied during the 2023 and 2024 dry seasons (May to September), a period when wildfires are more frequent in the region.

### 2.2. Participants

The CBMDF has approximately 2200 firefighters engaged in activities including wildfire suppression, urban firefighting, pre-hospital care, search and rescue, civil defense, training, and administrative duties (personal communication with Captain João Rafael). Firefighters who receive institutional specialization training in wildfire prevention and suppression are preferentially assigned to this activity during the dry season. Brigades are temporary workers who are active mostly during the dry season and exclusively perform wildfire-related activities in conservation units and parks. On average, about 150 brigades are available for these activities each year [19]. The questionnaire was administered to 150 military firefighters and 22 brigades.

### 2.3. Questionnaire

The questionnaire was pretested with 10 firefighters to address any lack of clarity and adjusted before being administered to the study participants. The final version covered sociodemographic information (gender, age, civil status, and education), years of firefighting experience, general health status, and smoking and alcohol habits (Table 1). Table 2 presents questions on institutional performance and risk perception regarding wildfire smoke exposure and RPE use. The risk perception questions 4, 5, 6, 8, 9, and 12 were ranked from 4 (indicating a higher perception) to 1 (indicating a lower perception). The study was approved by the Research Ethics Committee of the Faculty of Health Sciences, University of Brasília (CAAE 55389921.9.0000.0030). All participants signed an informed consent form prior to participation.

### 2.4. Statistical Analysis

The statistical analyses were performed using GraphPad Prism V 10.6.1. The parameters evaluated were age, education, years in the job, years as firefighters, alcohol consumption, institution performance, levels of concern, smoke has bothered, smoke can be harmful, risk in wildfires lower than structural, necessary to use RPE, and use RPE if provided. Age, years in the job or as a firefighter, and alcohol consumption were discrete values, while the others were assigned scores, as shown in Table 1 and Table 2. With the exception of age, the parameter distributions were not parametric (Shapiro–Wilk test). Correlations among parameters within each population were evaluated using Pearson correlation coefficients (r), and comparisons of the same parameter between the two populations were made using the Mann–Whitney test for nonparametric data or the Welch’s *t*-test for normal distribution. Significance was found at *p* ≤ 0.05. Reliability (internal consistency) of the questionnaire was assessed by calculating Cronbach’s alpha in IBM SPSS Statistics V.28, yielding a value of 0.41. The variance inflation factor (VIF) was below 2, indicating no multicollinearity among the variables.

## 3. Results

### 3.1. Sociodemographic and Habit Profiles

Only eight participants were women, all of whom were firefighters (Table 1). There was no significant age difference between the two groups (means of 37.5 and 40.6 years, respectively), with most firefighters aged 31–40 years (63.3%), followed by 41–50 years (18.7%), which was also the predominant age group among brigades (36.4%). Most firefighters and brigades were married (70.7 and 68.2%, respectively).

Firefighters had a significantly higher level of education than brigades (*p* < 0.0001), with 94.6% holding a college degree or postgraduate education, compared to 50% among brigades. No significant differences in years of wildfire suppression experience were found between the two groups (8.8 and 6.9 years, respectively), with about 45% of them working in this activity in the last five years. No brigade had more than 20 years of service. Almost half of the firefighters (47.3%) were specialists in wildfire prevention and suppression and worked exclusively in this area at the time of the study.

More than 90% of firefighters and all brigades reported no health problems. Among firefighters who did report health issues, hypertension and respiratory diseases were the most common. Fewer than 10% of participants smoked. Most firefighters (58%) consumed alcohol at least once a week, a higher percentage than among brigades (31.8%), although no significant difference in mean weekly consumption was found between the two groups (0.83 and 0.77 times/week, respectively).

### 3.2. Risk Perception and Use of Respiratory Protection Equipment

Brigades scored higher on the evaluation of institutional performance regarding employee health and safety during the wildfire suppression activity than firefighters (mean scores of 3.14 and 1.49, respectively; *p* < 0.0001). More than half of the firefighters rated CBMDF’s performance as low or very low, while 22.7% of brigades rated IBRAM similarly (Table 2). Most firefighters (70%) and half of the brigades reported receiving institutional guidance on smoke-exposure risks, and about 80% of participants reported a good understanding of the combustion reaction occurring during wildfires.

### 3.3. Risk Perception of Smoke Exposure During Wildfires

Most respondents reported a high level of concern regarding wildfire smoke exposure, although the brigade risk perception was higher than that of firefighters (mean scores of 3.64 and 2.46, respectively; *p* < 0.0001) (Table 2). Only two firefighters and no brigades reported no concern. All brigades and 96.7% of firefighters reported experiencing discomfort from smoke at least sometimes, with a significant difference in mean scores between the two groups (3.54 and 2.33, respectively; *p* < 0.0001).

The perception that smoke can be harmful to health was significantly higher among the brigades (mean score of 3.96 vs. 1.87; *p* < 0.0001). About 90% reported respiratory disorders as a consequence of smoke exposure. At least 80% of firefighters also reported headache/malaise/nausea and cancer, with lower percentages among brigades (50% and 36.4%, respectively). About 55% of firefighters and 36.4% of brigades believed that the smoke-exposure risk during wildfires is, or probably is, not lower than during structural fires, with similar risk-perception scores between the two groups.

### 3.4. Use of Respiratory Protection Equipment

More than 70% of respondents confirmed the need to use RPE during wildfires, with mean scores similar in the two groups, but only 31 firefighters and three brigades used RPE at the time of the study. Among these, 41.9% of firefighters and 66.7% of brigades adapted well to the equipment; two-thirds of firefighters and one-third of brigades reported discomfort; and 25% of firefighters and one-third of brigades reported improved health with RPE use. The main reason for non-use was a lack of equipment provision (53.8% of firefighters and 76.7% of brigades). Only two firefighters considered RPE unnecessary. About 12% of firefighters and 10.5% of brigades cited the lack of proven efficacy of available masks. More than 90% stated they would/probably use RPE if provided by the institution.

Finally, when asked whether they consider it important to assess firefighters’ exposure to smoke and the potential health risks during wildfire suppression, almost all participants answered yes.

### 3.5. Correlations Between the Investigated Parameters

**Figure 1:**
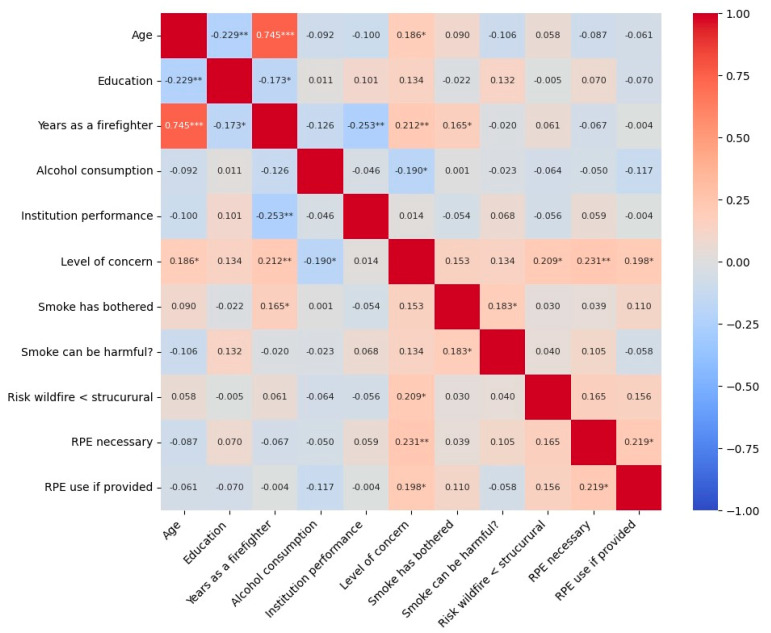
Pearson correlations between the parameters shown in Table 1 and Table 2 for the military firefighters. * *p* ≤ 0.05; ** *p* ≤ 0.01; *** *p* < 0.0001.

**Figure 2:**
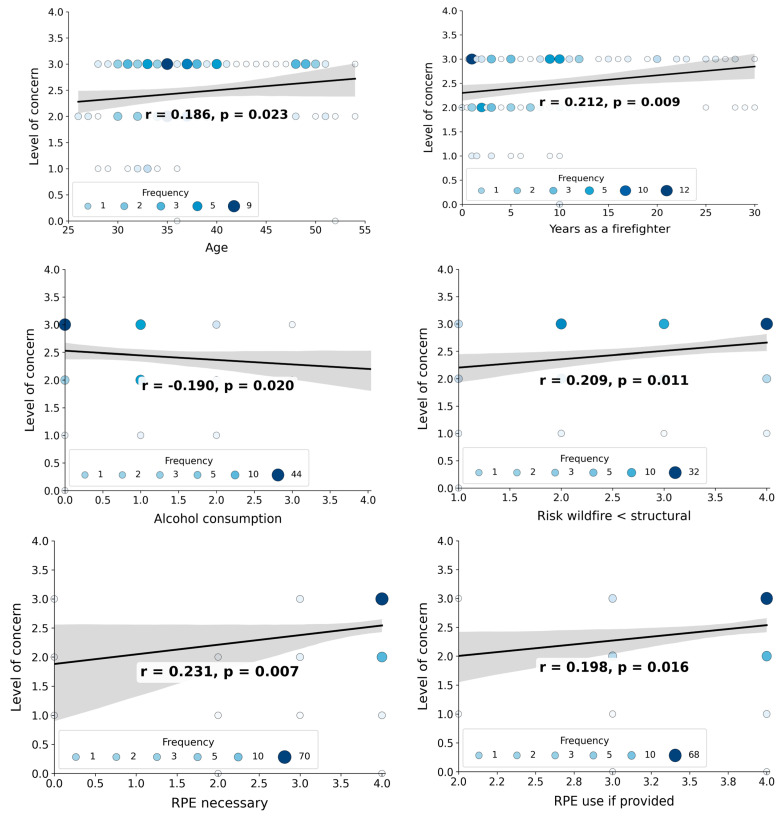
Plots of the significant correlations between the parameters investigated and the level of concern related to the exposure to smoke from wildfire among the military firefighters in the Federal District.

Figure 1 shows a heatmap of Pearson correlations (r) between the parameters listed in Table 1 and Table 2 for the military firefighters. Out of the 121 tested correlations, 13 were significant (*p* ≤ 0.05), although most had an r below 0.5. Figure 2 shows the significant correlation plots between six parameters and the level of concern regarding exposure to wildfire smoke.

Age correlated negatively with education (r = −0.229; *p* = 0.005) and positively with years of experience in wildfire suppression (r = 0.745; *p* < 0.0001) and with level of concern (r = 0.186; *p* = 0.023). Education correlates negatively with years of experience (r = −0.173; *p* = 0.034), which also correlates negatively with institution performance (r = −0.253; *p* = 0.002) and positively with level of concern (r = 0.212; *p* = 0.009) and with being bothered by the wildfire smoke (r = 0.165; *p* = 0.043). Being bothered by smoke also correlates with the perception that smoke can be harmful (r = 0.183; *p* = 0.025). Alcohol consumption correlated negatively with the level of concern (r = −0,190; *p* = 0.02), which also correlated with the perception that wildfire risk is not lower than structural risk (r = 0.209; *p* = 0.011), RPE necessary (r = 0.231; *p* = 0.007), and use it if provided (r = 0.198; *p* = 0.016), with the last two parameters correlating with each (r = 0.219; *p* = 0.011).

Fewer significant correlations were found among the forest brigades: age vs. time working (r = 0.3628, *p* = 0.0036), necessary to use RPE vs. education (r = 0.498, *p* = 0.0239) and vs. institution performance (r = −0.5829, *p* = 0.0056), and alcohol consumption vs. concern of smoke exposure (r = −0.4306, *p* = 0.0456). 

## 4. Discussion

Risk perception may vary according to sociodemographic characteristics such as gender, age, education, culture, length of professional experience, and geographic origin [12,13]. The population in this study is predominantly men aged 31 to 50 years, married or in stable relationships. Firefighters had a significantly higher educational level than that of brigades, which is expected, since a college degree has been mandatory to join the military firefighter service since 2011, a requirement that does not apply to brigades. Most participants have up to 10 years of experience working in wildfire suppression. Most participants reported no health problems, do not smoke, and consume little or no alcohol. One interesting result found in both groups is the negative correlation between the level of concern related to smoke exposure and alcohol consumption.

Sadler et al. [20] identified that risk perception is higher among professional firefighters than among volunteers and that this perception directly influences adherence to health prevention and safety practices. In the present study, brigades, who are not volunteers but employees of a governmental institution, have greater concern about wildfire smoke than military firefighters. More than 80% of firefighters held a college degree; however, education level was not associated with risk perception, a finding similar to that reported by Fialho et al. [17] in Portugal; meanwhile, Rodriguez-Garzón et al. [16] found that higher education was associated with higher risk perception in Ecuador. Among brigades, however, the need for RPE use was positively correlated with education level, a result not observed among firefighters.

A significant correlation was found between age and concern about wildfire smoke exposure among firefighters, but not among brigades. Furthermore, years of firefighting experience were correlated with concern about wildfire smoke and with being bothered by the smoke, but not with RPE use. Other studies showed similar or contradictory results. Fialho et al. [17] found that firefighters with less than five years of service had the lowest mean risk perception and identified that resistance to RPE use is higher among younger and less experienced Portuguese firefighters due to discomfort and the perception that it hinders work performance. According to Solle et al. [21], novice firefighters tend to be more concerned about immediate health and safety risks, whereas more experienced firefighters are more concerned about long-term effects, such as cancer. Bel-Latour and Granié [22] showed that younger French firefighters exhibit a higher frequency of risky behaviors, likely due to workplace socialization processes that reinforce masculine behaviors, which may lead to neglect of safety and health prevention. Conversely, Harrison et al. [23] found that older and more experienced firefighters have lower risk perception. According to these authors, younger firefighters may have greater access to information and, therefore, more knowledge about exposure risks, whereas older firefighters may rely more on practical experience, which does not always align with scientific research. In a study conducted in Australia, Sadler et al. [20] found that firefighters with longer service time, thus greater experience and training, demonstrated higher awareness and, consequently, higher risk perception, a result that was also found in the present study as discussed earlier. According to the authors, these firefighters tended to adopt safer behaviors, reinforcing the importance of risk perception in implementing effective safety strategies [20].

According to Maglio et al. [24], the main factors influencing non-use or improper use of RPE are firefighters’ personality traits, a strong focus on accomplishing the rescue objective that leads them to prioritize outcomes over safety, discomfort caused by RPE, and social pressure from peers not to use it. In the present study, although more than 90% of participants considered RPE use necessary, fewer than 20% actually used it; however, nearly all reported they would use it if it were provided by the institution. This suggests that RPE use is feasible and that resistance appears to be related to factors beyond discomfort alone, which most firefighters reported. Other studies have also reported low adherence to RPE use despite recognition of smoke-related risks, citing discomfort, difficulty breathing, or impaired communication [14,24,25]. According to Anderson et al. [14], both novice and experienced firefighters tend to adopt a “high-risk/high-reward” mindset, in which they believe the risks of the profession are worth taking because the benefits outweigh them.

The main health concerns for both groups in the present study are respiratory distress, headache/malaise/nausea, and cancer. In a study involving firefighters from Europe and Latin America, Carballo-Leyenda et al. [26] concluded that risk perception of cancer due to smoke exposure is independent of age and experience. In fact, more than 90% of firefighters in the present study believe that smoke exposure can cause cancer.

In Brazil, a study among military firefighters in Belo Horizonte, Minas Gerais, found that 92% of respondents considered the use of RPE necessary during wildfire suppression activities and emphasized the need to develop equipment specific to these activities; 90% expressed receptiveness to the future implementation of new RPE [18].

The need for RPE during wildfires, however, remains controversial. For years, the United States Forest Service (USFS) did not encourage respirator use because it was expected to reduce field work capacity due to increased breathing resistance [27]. Its use was recommended only when training, tactics, and monitoring fail to protect worker health and safety, as fewer than 5% of wildfire cases exceed Occupational Safety and Health Administration (OSHA) permissible exposure limits [27]. However, OSHA limits were based on predicted harm from inert dust, and, given that wildfire particulate matter (PM) is likely more harmful than particulate matter from other sources, safe exposure limits may underestimate potential health effects [28]. In September 2025, the USFS published new guidance acknowledging, for the first time, that masks can protect firefighters from harmful particles in wildfire smoke. The guidance promotes the “use of a properly-fitted N95 respirator on a clean-shaven face with a successful fit test”, which “will remove at least 95 percent of airborne particles,” (https://fas.org/publication/n95-firefighters/ accessed on 8 May 2026). Part of the resistance to RPE use stems from the inadequate ergonomics of available models, which hinder breathing during intense physical exertion [24]. Overall, firefighters still face considerable uncertainty regarding the efficiency and appropriateness of available protective equipment [23].

About 15% of firefighters reported not using RPE due to a lack of proven efficacy. According to Filiberti et al. [28], current guidelines discourage the use of respirators because they filter respiratory irritants but not all hazardous components of smoke. The discomfort reported by approximately 10% of firefighters when using respiratory masks is consistent with the literature. Many wildland firefighters report breathing difficulties, communication interference, and sensations of suffocation from masks, due to intense physical exertion and heat exposure [28].

All forest brigades reported improved performance or well-being from RPE use, compared with only 25% of firefighters, indicating a significant difference in perception that may be related to organizational culture or the type of RPE used. It is noteworthy that the few professionals who reported using RPE purchased the equipment on their own initiative and with personal resources, without institutional guidance or technical information.

When asked whether they would use RPE if it were provided, more than 90% of participants responded “yes” or “probably yes,” indicating that, in principle, there is no resistance. Therefore, organizational support and encouragement are crucial for RPE adherence [17]. When equipment is provided, and institutions offer adequate support and training, adherence increases significantly, even in challenging work environments that require intense physical effort, such as wildfire suppression [29,30]. Resistance to RPE use decreases considerably when professionals perceive legitimacy and encouragement from their institution [17]. Regular provision and standardization of protective equipment for wildland firefighters increases spontaneous acceptance and risk perception [21].

In the present study, about half of the participants agreed that the risk of smoke exposure is lower during wildfires than during structural fires, and there was a positive correlation between the perception that the risk from wildfire smoke is not lower and the concern about wildfire smoke. The perception that wildfire smoke is less dangerous is not entirely incorrect, since structural fire smoke can cause severe health damage more rapidly, including immediate life-threatening effects, whereas wildfire smoke tends to cause more severe long-term (chronic) effects, such as cancer. Vegetation burning releases toxic compounds such as benzene, toluene, ethylbenzene, xylenes (BTEX), formaldehyde, acrolein, and fine particulate matter (PM up to 2.5 µm) [1], whereas structural fire smoke often also contains compounds from burning plastics, paints, industrial products, and synthetic materials [2].

The symptoms described by participants as resulting from smoke exposure were also reported in other studies [18,25,26,31,32]. Headache, malaise, and nausea are acute symptoms associated with wildfire smoke exposure, which contains irritant gases and toxic compounds, including carbon monoxide, benzene, and PAHs [1,18]. Cancer risk was cited by most firefighters, indicating a level of chronic risk perception consistent with studies linking continuous smoke exposure to increased cancer risk [33,34]. Occupational cancer risk has also been reported as the main health concern among both novice and experienced firefighters in other studies [21,23]. Fewer than 40% of brigades mentioned this risk, possibly because of their shorter service in fire suppression.

Most CBDF firefighters rated institutional performance as low or very low, and these ratings were negatively correlated with years of wildfire suppression experience. Among the brigades, fewer than 30% rated IBRAM’s performance as low/very low, possibly due to the temporary nature of their work and fear of expressing dissatisfaction stemming from a lack of job stability.

The main contribution of this study was to include contracted brigades in the survey and to conclude that they perceive a higher risk of exposure to wildfire smoke than the military firefighters. This is the first time that these two populations have been compared.

This study has some limitations. First, it is a cross-sectional study based on a questionnaire applied at a single point in time, which precludes causal inferences among risk perception, RPE use, and other investigated factors. Additionally, participants were selected by convenience sampling and may not represent the entire population, and the study relies on self-reported data, which may be subject to recall bias and socially desirable responses. The number of brigade participants is much lower than that of firefighters, which may compromise the conclusions when the groups are compared. Finally, the study was conducted in a specific region of Brazil and may not be generalized to other regions of the country or the world.

## 5. Conclusions

Overall, the participants in this study have a high level of education, particularly among firefighters, and most are experienced in wildfire suppression. Both military firefighters and forest brigades demonstrate risk perception regarding health risks from exposure to wildfire smoke, and most recognize that continuous, frequent exposure poses a threat and are aware of both acute and chronic effects. However, the brigades’ risk perception is significantly higher than that of the firefighters.

Even so, a gap is observed between this risk perception and practical preventive and protective behaviors. Few firefighters and brigades use RPE, mainly due to the lack of institutional provision. For safe behaviors to effectively occur, not only is individual awareness required, but also effective institutional health protection policies, including guidance, provision of appropriate equipment, training, and occupational health monitoring of exposed professionals. Most military firefighters are dissatisfied with their institution’s performance in occupational health and safety management, underscoring the need to improve training and institutional health and safety programs.

## Figures and Tables

**Table 1:** Sociodemographic profile and other parameters of the participants (military firefighters, N = 150; brigades, N = 22).

Parameter (Score)	Military Firefighters, n (%)	Brigades, n (%)
1. Men	142 (94.7)	22 (100.0)
2. Age, years ^a^		
Up to 30	19 (12.7)	4 (18.2)
31 to 40	95 (63.3)	6 (27.3)
41 to 50	28 (18.7)	8 (36.4)
>50	8 (5.3)	4 (18.2)
Mean ± sd	37.5 ± 6.8	40.6 ± 9.2
2. Civil status		
Married	106 (70.7)	15 (68.2)
Single	31 (20.7)	6 (27.3)
Divorced	12 (8.0)	1 (4.5)
Widow	1 (0.7)	0
3. Education		
Elementary (1)	0	2 (9.0)
High school (2)	8 (5.3)	9 (41.0)
College (3)	119 (79.3)	9 (41.0)
Graduate school (4)	23 (15.3)	2 (9.0)
Mean ± sd	3.1 ± 0.44 *	2.5 ± 0.80 *
4. Years ^a^ as a firefighter ^b^ or brigade		
Up to 5	67 (44.7)	10 (45.5)
6 to 10	40 (26.7)	6 (27.3)
11 to 20	26 (17.3)	6 (27.3)
21 to 25	8 (5.3)	0
26 to 30	9 (6.0)	0
Mean ± sd	8.8 ± 7.8	6.9 ± 5.3
5. Health problems		
None	137 (91.3)	22 (100.0)
Hypertension	5 (3.3)	0
Respiratory diseases	3 (2.0)	0
Back pain	2 (1.3)	0
Diabetes	1 (0.7)	0
Others	2 (1.3)	0
6. Smoke	12 (8.0)	1 (4.5)
7. Alcohol consumption, times/week		
0	65 (43.3)	7 (31.8)
1	58 (38.7)	13 (59.1)
2	19 (12.7)	2 (9.1)
3	6 (4.0)	0
4/5	2 (1.3)	0
Mean ± sd	0.83 ± 0.95	0.77 ± 0.61

sd = standard deviation; ^a^ continuous variable; ^b^ in suppression of wildfire; * significant difference at *p* < 0.0001 (Mann–Whitney test).

**Table 2:** Risk perception of exposure to the smoke of forest fires and the use of respiratory protection equipment (RPE) (military firefighters, N = 150; brigades, N = 22).

Parameter (Score)	Military Firefighters, n (%)	Brigades, n (%)
1. How do you judge the performance of your institution?		
Good/very good (4)	11 (7.3)	10 (45.5)
Acceptable (3)	48 (32.0)	6 (27.3)
Low/very low (2)	89 (59.3)	5 (22.7)
Don’t know (1)	2 (1.3)	1 (4.5)
Mean score ± sd	1.49 ± 0.65 *	3.14 ± 0.94 *
2. Was advised by the institution about the risk	105 (70.0)	11 (50.0)
3. Understand the combustion reaction	120 (80.0)	18 (81.2)
4. Levels of concern about the smoke		
High (4)	83 (55.3)	15 (68.2)
Average (3)	55 (36.7)	6 (27.3)
Low (2)	10 (6.7)	1 (4.5)
No concern (1)	2 (1.3)	0
Mean score ± sd	2.46 ± 0.68 *	3.64 ± 0.58 *
5. Smoke has bothered you		
Always (4)	60 (40.0)	12 (54.5)
Sometimes (3)	85 (56.7)	10 (45.4)
Never (2)	4 (2.7)	0
Don’t remember/never (1)	1 (0.7)	0
Mean score ± sd	2.33 ± 0.65 *	3.54 ± 0.51 *
6. Smoke can be harmful to health		
Frequently (4)	130 (86.7)	21 (95.4)
Occasionally (3)	20 (13.3)	1 (4.6)
Rarely (2) Not at all (1)	0 0	0 0
Mean score ± sd	1.87 ± 0.34 *	3.96 ± 0.21 *
7. Symptoms caused by smoke		
Respiratory distress	137 (91.3)	20 (90.9)
Headache/malaise/nausea	125 (83.3)	11 (50.0)
Cancer	137 (91.3)	8 (36.4)
Other symptoms	53 (35.3)	6 (27.3)
8. Risk from smoke exposure in wildfire is lower than in structural fires		
No (4)	45 (30.0)	4 (18.2)
Probably no (3)	38 (25.3)	4 (18.2)
Probably yes (2)	44 (29.3)	8 (36.4)
Yes (1)	23 (15.3)	6 (27.3)
Mean score ± sd	2.71 ± 1.05	2.27 ± 1.08
9. Necessary to use RPE in forest fires		
Yes (4)	111 (74.0)	17 (77.3)
Probably yes (3)	21 (14.0)	3 (13.6)
Probably no (2)	13 (8.7)	1 (4.5)
No (1)	5 (33.0)	1 (4.5)
Mean score ± sd	3.67 ± 0.86	3.64 ± 0.79
10. Use RPE	31 (20.7)	3 (13.6)
Good adaptation ^a^	13 (41.9)	2 (66.7)
Uncomfortable ^a^	21 (67.7)	1 (33.3)
Felt better when used it ^a^	8 (25.8)	1 (33.3)
11. Why don’t use ^b^		
Not provided	64 (53.8)	14 (73.7)
Makes breathing difficult	16 (13.5)	0
There is no proven efficacy	14 (11.8)	2 (10.5)
Not necessary	2 (1.7)	0
Not informed	23 (19.3)	3 (15.8)
12. Would use RPE if provided		
Yes (4)	110 (73.3)	18 (81.8)
Probably yes (3)	38 (25.3)	3 (13.6)
Probably no (2)	2 (1.3)	1 (4.5)
No (1)	0	0
Mean score ± sd	3.7 ± 0.48	3.6 ± 0.79
13. Important to assess firefighters’ exposure and potential health risks		
Yes	148 (98.7)	22 (100.0)
No	0	0
Don’t know	2 (1.3)	0

sd = standard deviation; ^a^ percent related to those who use PRE: 31 military firefighters and 3 brigades; ^b^ percent related to those who do not use PRE: 119 military firefighters and 19 brigades; * significant difference; *p* < 0.0001. The Mann–Whitney test was used for all cases except for question 4 (Welch’s *t*-test).

## Data Availability

The original contributions presented in this study are included in the article. Further inquiries can be directed to the corresponding author.

## References

[1] Adetona O., Reinhardt T.E., Domitrovich J., Broyles G., Adetona A.M., Kleinman M.T., Ottmar R.D., Naeher L.P. (2016). Review of the health effects of wildland fire smoke on wildland firefighters and the public. Inhal. Toxicol..

[2] Fent K.W., Eisenberg J., Snawder J., Sammons D., Pleil J.D., Stiegel M.A., Dalton J. (2014). Systemic exposure to PAHs and benzene in firefighters suppressing controlled structure fires. Ann. Occup. Hyg..

[3] Urbanski S.P., Hao W.M., Baker S. (2009). Chemical composition of wildland fire emissions. Dev. Environ. Sci..

[4] Mayer A.C., Fent K.W., Wilkinson A., Chen I.C., Kerber S., Smith D.L., Kesler R.M., Horn G.P. (2022). Characterizing exposure to benzene, toluene, and naphthalene in firefighters wearing different types of new or laundered PPE. Int. J. Hyg. Environ. Health.

[5] Gould C.F., Heft-Neal S., Johnson M., Aguilera J., Burke M., Nadeau K. (2024). Health effects of wildfire smoke exposure. Ann. Rev. Med..

[6] Barboni T., Cannac M., Pasqualini V., Simeoni A., Leoni E., Chiaramonti N. (2010). Volatile and semi-volatile organic compounds in smoke exposure of firefighters during prescribed burning in the Mediterranean region. Int. J. Wildland Fire.

[7] Cherry N., Fedun M., Galarneau J.M., Senkevics D., Zadunayski T. (2025). Health effects of repeated exposures during wildland firefighting: A data-linkage cohort study from Alberta, Canada. Ann. Work Expo. Health.

[8] Sritharan J., Kirkham T.L., MacLeod J., Marjerrison N., Lau A., Dakouo M., Logar-Henderson C., Norzin T., DeBono N.L., Demers P.A. (2022). Cancer risk among firefighters and police in the Ontario workforce. Occup. Environ. Med..

[9] Hunter A.A., Unosson J., Bosson J.A., Langrish J.P., Pourazar J., Raftis J.B., Miller M.R., Lucking A.J., Boman C., Nyström R. (2014). Effect of wood smoke exposure on vascular function and thrombus formation in healthy fire fighters. Part. Fib. Toxicol..

[10] Esteves F., Madureira J., Costa C., Pires J., Barros B., Alves S., Vaz J., Oliveira M., Slezakova K., Fernandes A. (2025). Occupational exposure to wildland firefighting and its effects on systemic DNA damage. Int. J. Hyg. Environ. Health.

[11] International Agency for Research on Cancer (2026). Agents Classified by the IARC Monographs. Volumes. 1–137. https://monographs.iarc.who.int/agents-classified-by-the-iarc.

[12] Hampel J. (2006). Different concepts of risk—A challenge for risk communication. Int. J. Med. Microb..

[13] Prati G., Pietrantoni L., Saccinto E., Kehl D., Knuthc D., Schmidt S. (2013). Risk perception of different emergencies in a sample of European firefighters. Work.

[14] Anderson D.A., Harrison T.R., Yang F., Wendorf Muhamad J., Morgan S.E. (2017). Firefighter perceptions of cancer risk: Results of a qualitative study. Am. J. Ind. Med..

[15] Louzado-Feliciano P., Santiago K.M., Paule L., Rivera G., Solle N.S., Miric M., Perez-Then E., Caban-Martinez A.J. (2022). Perceptions of occupational cancer risk and prevention among Dominican Republic firefighters: A qualitative study. J. Occup. Environ. Med..

[16] Rodriguez-Garzón I., Fiestas M.M., Padial A.D., Ruiz V.L. (2016). Perception of occupational risk of firefighters in Quito (Ecuador). Fire Technol..

[17] Fialho M., Nunes S., Gamelas C.A. (2024). Risk perception, safety behavior and work accidents: Assessment and relations in a sample of Portuguese firefighters. Fire Technol..

[18] Silva R.A., Paiva M.J.N., Martins I. (2025). Perception of health risks and occupational exposure to forest fires among military firefighters in Belo Horizonte, Minas Gerais. Vigiles.

[19] Instituto Brasília Ambiental (2025). Edital nº 3, de 16 de Maio de 2025. Dispõe Sobre a Abertura de Processo Seletivo Para Contratação de Brigadistas. Brasília. https://www.ibram.df.gov.br/documents/26676051/26679862/edital_no_03_de_16_de_maio_de_2025.pdf.

[20] Sadler P., Holgate A., Clancy D. (2007). Is a contained fire less risky than a going fire? Career and volunteer firefighters’ perception of risk. Austr. J. Emerg. Manag..

[21] Solle N.S., Caban-Martinez A.J., Levy R.A., Young B., Lee D., Harrison T., Kobetz E. (2018). Perceptions of health and cancer risk among newly recruited firefighters in South Florida. Am. J. Ind. Med..

[22] Bel-Latour L., Granié M.-A. (2022). The influence of the perceived masculinity of an occupation on risk behavior: The case of firefighters. Saf. Sci..

[23] Harrison T.R., Muhamad J.W., Malova E. (2022). Firefighters and cancer: A review of the current state of cancer incidences and recent trends in risk perception and risk reduction efforts. Med. Res. Arch..

[24] Maglio M.A., Scott C., Davis A.L., Allen J., Taylor J.A. (2016). Situational pressures that influence firefighters’ decision making about personal protective equipment: A qualitative analysis. Am. J. Health Behav..

[25] Zadunayski T., Broznitsky N., Lichty D., Cherry N. (2024). Perceptions of exposure and mask use in wildland firefighters. Toxics.

[26] Carballo-Leyenda B., Villa-Vicente J.G., Delogu G.M., Rodríguez-Marroyo J.A., Molina-Terrén D.M. (2022). Perceptions of heat stress, heat strain and mitigation practices in wildfire suppression across Southern Europe and Latin America. Int. J. Environ. Res. Public Health.

[27] Sharkey B. (1997). Health hazards of smoke: Recommendations of the consensus conference April 1997. Proceedings of USDA/USFS Consensus Conference.

[28] Filiberti A.A., Davis S.C., Spano S.J. (2025). Smoke Exposure and Respirator Use Among Wildland Firefighters: A Narrative Review. Wild. Environ. Med..

[29] Daniels R.D., Bertke S., Dahm M.M., Yiin J.H., Kubale T.L., Hales T.R., Baris D., Zahm S.H., Beaumont J.J., Waters K.M. (2015). Exposure-response relationships for select cancer and non-cancer health outcomes in a cohort of U.S. firefighters from San Francisco, Chicago and Philadelphia (1950–2009). Occup. Environ. Med..

[30] Safak I., Okan T., Karademir D. (2023). Perceptions of Turkish forest firefighters on in-service trainings. Fire.

[31] Millet B. (2020). Designing an occupational exposure report for aircraft rescue and firefighting. Proc. Hum. Fact. Ergon. Soc. Ann. Meet..

[32] Shah N.N., Steinberg M.B., Caban-Martinez A.J., Austin E., Burgess J.L., Hollerbach B.S., Edwards D.L., Black T.M., Black K., Hinton K.M. (2023). Prevalence and predictors of skin cancer screening among sample of United States volunteer firefighters. Am. J. Ind. Med..

[33] McClanahan K., Sanchez P., Gant K., Joyce J., Braun A. (2024). Perceptions of preventable cancer burden among US-based firefighters: A mixed methods cross-sectional study. J. Nut. Educ. Behav..

[34] Eun Oh H.E., Kim J.S., Woo H., Ham S. (2022). Associations between awareness of the risk of exposure to pollutants occurring at fire scenes and health beliefs among metropolitan firefighters in the Republic of Korea. Int. J. Environ. Res. Public Health.

